# Circular RNA hsa_circ_0000511 Improves Epithelial Mesenchymal Transition of Cervical Cancer by Regulating hsa-mir-296-5p/HMGA1

**DOI:** 10.1155/2021/9964538

**Published:** 2021-05-27

**Authors:** Jia Xie, Qian Chen, Ping Zhou, Wenli Fan

**Affiliations:** ^1^Department of Gynecology, Hospital of Chengdu University of Traditional Chinese Medicine, Chengdu, China; ^2^Wuhan Children's Hospital (Wuhan Maternal and Child Healthcare Hospital), Tongji Medical College, Huazhong University of Science & Technology, Wuhan, China

## Abstract

As the second largest gynecological cancer, cervical cancer has been widely reported in recent years in which circular RNA is involved in the disease process. We earlier found that the expression of hsa_circ_0000511 in cervical cancer cells increased significantly, but its role in the process of cervical cancer is not clear. The purpose of this study is to explore its possible mechanisms in cervical cancer. Quantitative reverse transcription polymerase chain reaction (qRT-PCR), cell counting kit-8 assay, Transwell test, cell transfection, RNA pull-down assay and dual-luciferase reporter assay, and Western blot analysis were used to detect the expression and distribution of hsa_circ_0000511 in SiHa and HeLa cells, the ability of invasion and proliferation, and the modulated relationships between hsa_circ_0000511 and hsa-mir-296-5p, hsa-mir-296-5p, and HMGA1. hsa_circ_0000511 had the highest expression in SiHa and HeLa cells, and the expression in the cytoplasm was significantly higher than that in the nucleus, and its expression was not affected by RNase R. When hsa_circ_0000511 was silenced, its expression in SiHa and HeLa cells was significantly decreased; the proliferation, invasion, and migration abilities of the two kinds of cells were significantly enhanced; and the protein expression of E-cadherin was significantly upregulated, while the protein expression of N-cadherin was significantly downregulated. The expression of hsa-mir-296-5p was lower in SiHa and HeLa cells; however, its expression was increased when hsa_circ_0000511 was inhibited and decreased when hsa_circ_0000511 was overexpressed, so did the ability of proliferation, invasion, and migration and the protein expression of E-cadherin. Interestingly, the protein expression of HMGA1 also changed in these two cells when hsa-mir-296-5p was inhibited or overexpressed. Our results indicate that the upregulated hsa_circ_0000511 can inhibit the proliferation, invasion, and migration of SiHa and HeLa cells by regulating hsa-mir-296-5p/HMGA1, suggesting that the hsa_circ_0000511/hsa-mir-296-5p/HMGA1 pathway may be a potential target for the treatment of cervical cancer.

## 1. Introduction

Cervical cancer, a common malignant tumor, is a major health challenge for women around the world. Only in the year 2018, there were about 570000 new cervical cancer patients in the world, and more than 311000 patients died [1]. It is gratifying that cervical cancer has been confirmed to be mainly caused by high-risk subtypes of human papillomavirus (HPV) [2], and early screening and vaccination are effective measures to prevent cervical cancer [3–5]. However, the molecular mechanism of invasion and metastasis of cervical cancer is still unclear.

Circular RNAs (circRNAs) are endogenous RNAs, which are formed by reverse splicing of linear precursor mRNA [6]. However, circRNA can resist the degradation of exonuclease RNase R, which is more stable than linear mRNAs [7]. Some studies have found that some circRNAs can be highly specifically expressed in some cell types or at a certain stage of cell proliferation [8, 9]. Meanwhile, circRNAs may have the function of “sponge adsorption” of microRNAs and act as competitive endogenous RNAs to adsorb microRNAs, so as to regulate gene expression by binding proteins [10, 11]. Therefore, circRNA plays an important role in the pathogenesis of cancer, including cervical cancer. In fact, recent studies have found that some circRNAs, such as circAMOTL1 [12], circNFATC3 [13], circNRIP1 [14], circRNA-AKT1 [15], circAMOTL1 [16], and so on [17–19], are involved in the occurrence and development of cervical cancer.

MicroRNAs (miRNAs) with a size of 17-25 nucleotides have been confirmed to play roles in almost all processes of tumor biology, such as proliferation, invasion/metastasis, angiogenesis, and apoptosis [20]. Some miRNAs are upregulated in some tumors and act as promoters of cancer progression by silencing tumor suppressors, such as apoptosis-related genes. Therefore, many researchers believe that miRNA expression level analysis can be used as a biomarker for cancer diagnosis and progression [21]. Previous studies have found that some miRNAs such as miR-10a, miR-21, miR-19, and miR-146a play a role in the growth, migration, and invasion of cervical cancer cells, while miR-372, miR-214, and miR-218 have inhibitory effects on cervical cancer cells [21]. It is worth noting that a large number of recent studies have found that miRNAs are regulated by circRNAs and involved in the progression of cervical cancer, such as circNFATC3 which promotes the development of cervical cancer through sponging miR-9-5p [13], circNRIP1 which regulates the PTP4A1/ERK1/2 pathway through sponging miR-629-3p and promotes the migration and invasion of cervical cancer [14], and circ_0000520 sponge miR-146b-3p released PAX5 and expressed the progression of cervical cancer in vivo and in vivo [22]. In this study, we also found an abnormal expression of circRNA (circ_0000511) in cervical cancer cells (HcerEpic cells<SiHa<C-33A<HeLa<CaSki<SW756) by GEOanalysis (GSE113696) and then predicted its target by bioinformatics methods and verified it in vitro and in vivo.

## 2. Materials and Methods

### 2.1. Cell Culture

Human cervical epithelial immortalized cell lines H8 and C-33A were purchased from BeNa culture collection (Suzhou, Jiangsu, China), while the SiHa and HeLa cells were obtained from the cell bank of the Chinese Academy of Sciences (Shanghai, China). The first two were cultured in RPMI1640 medium (Gibco, Invitrogen, Waltham, MA, USA) containing 10% fetal bovine serum, and the second two were cultured in Dulbecco's modified Eagle medium (DMEM, Gibco) containing 10% fetal bovine serum (Gibco), 2 mM L-glutamine, and 1% penicillin/streptomycin. These cells were cultured at 37°C in a moist atmosphere (Thermo Fisher Scientific, Waltham, MA) with 5% CO_2_.

### 2.2. Quantitative Real-Time Polymerase Chain Reaction (qRT-PCR) Analysis

Total RNAs of these cells were extracted with GeneJET RNA purification kits (Thermo Fisher Scientific) according to the instructions. The reverse transcriptase was executed with high-capacity cDNA reverse transcription kits (Thermo Fisher Scientific) based on the manufacturer's manual and amplified with SYBR Premix DimerEraserTM (TaKaRa, Japan) in a real-time PCR Detection System (Bio-Rad, Hercules, CA). Triplicate experiments were performed with the specific primer sequences of hsa_circ_0000511, RPPH1, miR-296-5p, high-mobility group A1 (HMGA1), and GAPDH listed in Table 1. The relative expressions were normalized and calculated by the 2^−*ΔΔ*Ct^ method.

### 2.3. RNase R Assay

The RNA of SiHa and HeLa cells was isolated with RNeasy system. Then, the RiboMinus kit (Invitrogen, Carlsbad, California, USA) was used to perform our first and second biological replicate with 60 *μ*g and 20 *μ*g total RNA, respectively [11]. The diluted sample was denatured by heating (70°C) and cooled (40°C), then mixed with 10× RNase R buffer (1.7 *μ*L) at 40°C for 1 h. Each group was provided with three multiple wells. To assess the stability of hsa_circ_0000511 and *RPPH1* human mRNA, the expression levels were determined by using qRT-PCR.

### 2.4. Cell Proliferation Assay

The proliferation of transfected SiHa and HeLa cells was detected with a cell counting kit-8 (CCK-8; Beyotime, Shanghai, China) assay. In brief, SiHa and HeLa cells in the logarithmic growth phase were seeded in a 96-well plate with a cell density of 1 × 10^4^ cells/well. Cells were transfected with corresponding recombinant plasmids [17, 24] (Table 2) for 0, 12, 24, and 48 h, respectively. Each group was provided with three multiple wells. At each time point, 10 *μ*L CCK8 solution was added to each well and incubated at 37°C for 2 h; then, the absorbance was measured at 450 nm.

### 2.5. Transwell Assay

SiHa and HeLa cells were transfected with control, si-circ, si-circ-NC, miRNA mimics, miRNA NC, or overexpressed-HMGA1 for 24 hours. 1 × 10^5^ transfected cells were seeded in a serum-free DMEM in the upper part of a Transwell chamber while the lower Transwell chamber was with DMEM containing 10% fetal bovine serum. After incubation for 24 hours, the migrated or invasion cells were fixed with 4% paraformaldehyde solution for 10 minutes, then stained with 0.5% crystal violet for 25 minutes, and the cells were counted under the microscope. It should be noted that the upper layer of Transwell chambers was precoated with a 0.8% matrix in invasion experiment, but not in migration experiment. Six fields were randomly selected from each well for cell counting, and the average value was calculated. Each group was provided with three multiple wells, and the average values of the three wells were used for statistical analysis.

### 2.6. Dual-Luciferase Reporter Assay

The binding sites of hsa_circ_0000511 or HMGA1 in miR-296-5p were predicted with the ciecBank, Circinteractome, and starBase databases. To validate the interplay between circ_0000511 and miR-296-5p, miR-296-5p, and HMGA1, the sequences of wild-type (circRNA-wt) or mutant hsa_circ_0000511 (circRNA-mut), wild-type or mutant HMGA1 3′-UTR, miRNA mimics, and miRNA NC were cloned into the pGL3 vector (Promega, Wisconsin, USA) to form recombinant plasmids, respectively. The SiHa and HeLa cells were seeded into 96-well plates and cotransfected with the above plasmid. Each group was provided with three multiple wells. After transfection for 48 h, the fluorescence intensity was detected by dual-luciferase reporter assay system (Promega) according to the manufacturer's protocols.

### 2.7. Nude Mouse Tumorigenicity Assay

Transfected si-circRNA or si-circRNA+miRNA mimics and normal SiHa and HeLa cells (5 × 10^5^) were subcutaneously injected into the right side of 4-week-old thymus-free nude mice (*n* = 5/group). Four weeks later, the nude mice were killed by cervical spondylectomy, and the tumor tissues were taken out for photography. Then, the tumor was cut into two parts: one part was fixed with 4% paraformaldehyde solution for immunohistochemical staining, and the other part was cryopreserved in liquid nitrogen for protein detection. Animal experiments are approved by the Animal Protection and Utilization Committee.

### 2.8. Immunohistochemistry

The fixed tumor tissue was dehydrated and embedded, then cut into 5 *μ*m slices, and then repaired with citric acid (pH = 6.0). Then, antiproliferating cell nuclear antigen (PCNA) (ab92552, 1 : 400, Abcam) was incubated at 4°C overnight, and the second antibody was incubated at 37°C for 30 min. Subsequently, the sections were stained with hematoxylin after color development with a DAB kit (AR1022, Boster Biological Technology Co., Ltd., Wuhan, China). And the average optical density of each sample was determined by Image-Pro Plus 6.0 (Media Cybernetics, USA).

### 2.9. Western Blot Analysis

Total proteins of each cultured cell were extracted with RIPA Lysis Buffer (Applygen Technologies Inc., Beijing, China). After the protein concentration was determined by BCA kit (MultiSciences, Hangzhou, China), the target proteins were classified by 15% or 8% SDS-PAGE (MultiSciences) and then transferred to PVDF membranes (Millipore, Darmstadt, Germany) and blocked with 5% skimmed milk powder at 25°C for 90 minutes. The membranes were incubated overnight at 4°C with primary antibodies, anti-E-Cadherin (ab40772, 1 : 20000), anti-N-Cadherin (ab76011, 1 : 10000), anti-HMGA1 (ab168260, 1 : 1000), and anti-GAPDH (ab8245, 1 : 5000) (Abcam, Cambridge, UK). The membranes were then incubated with HRP-conjugated secondary antibodies (1 : 1000, MultiSciences) at 25°C for 90 min, and the bands were visualized by a chemiluminescence.

### 2.10. Statistical Analysis

All data were presented as the mean ± standard deviation (SD), the differences were analyzed by an unpaired two-sided *t*-test or one-way analysis of variance, and a value of *p* < 0.05 was regarded as statistically significant.

## 3. Results

### 3.1. hsa_circ_0000511 Was Highly Expressed in SiHa and HeLa Cells and Mainly Located in the Cytoplasm

**Figure 1:**
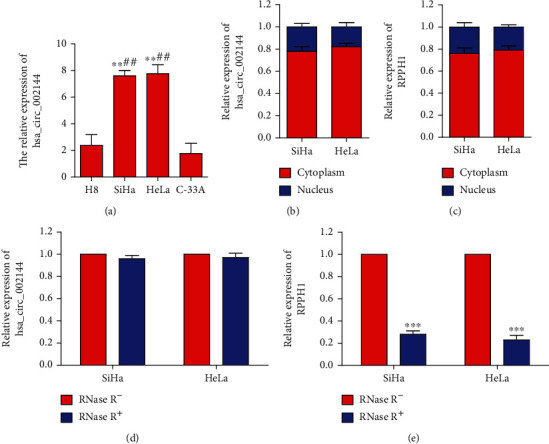
hsa_circ_0000511 was highly expressed in SiHa and HeLa cells and mainly located in the cytoplasm. The expression levels of hsa_circ_0000511 in SiHa and HeLa cells were significantly higher than those in H8 and C-33A cells (a) (mean ± SD; the data were analyzed by one-way analysis of variance; vs. H8, ^∗∗^*p* < 0.01; vs. C-33A, ^##^*p* < 0.01). hsa_circ_0000511 (b) and its gene symbol (*RPPH1*) (c) were mainly expressed in the cytoplasm in SiHa and HeLa cells. hsa_circ_0000511 can be resistant to RNase R^+^ (d), while *RPPH1* is not (e) (mean ± SD; the data were analyzed by an unpaired two-sided *t*-test; ^∗∗∗^*p* < 0.001).

In the early stage, we found that the expression of hsa_circ_0000511, also named hsa_circ_002144, was significantly upregulated in cervical cancer cells (HcerEpic cells<SiHa<C-33A<HeLa<CaSki<SW756). To verify this result, we used qRT-PCR to detect the expression of hsa_circ_0000511 in different cervical cancer cell lines. The results showed that the expression levels of hsa_circ_0000511 in SiHa and HeLa cells were higher than those in H8 and C-33A cells (Figure 1(a)). Therefore, in the next study, we used SiHa and HeLa cells as research vectors to continue to detect the distribution of SiHa and HeLa cells. As shown in Figures 1(b) and 1(c), the expression levels of hsa_circ_0000511 and its gene symbol (*RPPH1*) were mainly expressed in the cytoplasm in SiHa and HeLa cells, respectively. Meanwhile, we digested them with RNase R^+^ and detected their resistance to RNase R^+^. The results showed that the expression levels of hsa_circ_0000511 were not changed significantly in SiHa and HeLa cells treated with RNase R^+^ (Figure 1(d)), but those of *RPPH1* were decreased significantly (Figure 1(e)).

### 3.2. Inhibition of hsa_circ_0000511 Significantly Promoted the Invasion and Migration of SiHa and HeLa Cells

**Figure 2:**
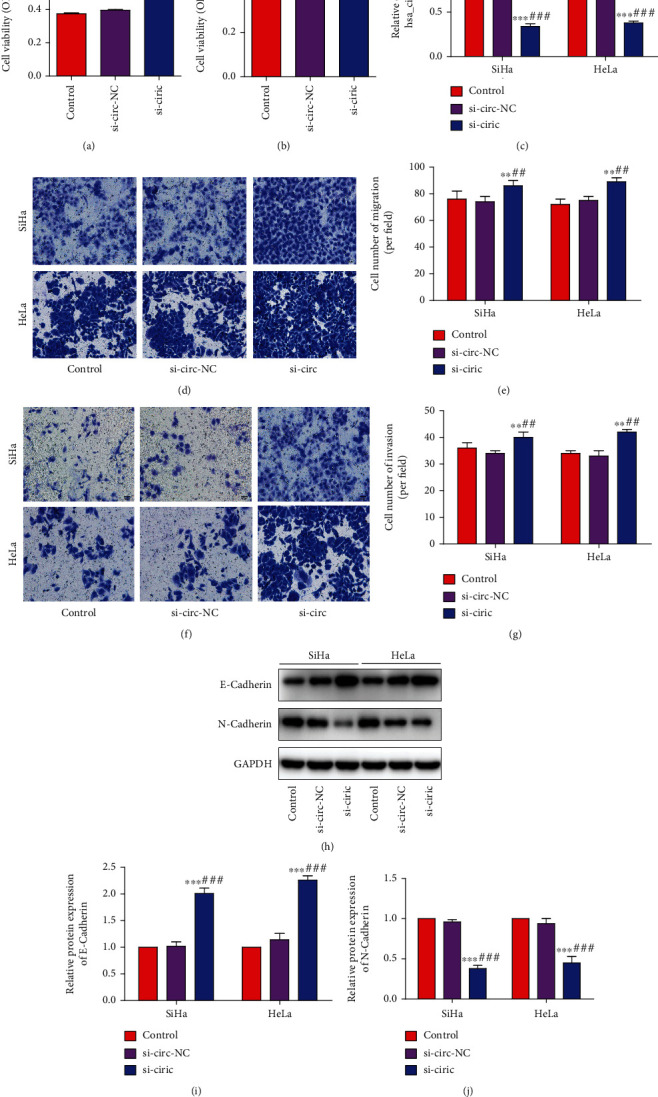
Inhibition of hsa_circ_0000511 significantly promoted the invasion and migration of SiHa and HeLa cells. The proliferation of SiHa (a) and HeLa (b) cells was increased when hsa_circ_0000511 was silenced; naturally, the expression of hsa_circ_0000511 was significantly decreased in the silenced cells (c). Moreover, when hsa_circ_0000511 was silenced, the invasion and migration abilities of SiHa (d) and HeLa (f) cells were also significantly enhanced; that is, the number of cells in lower Transwell chamber was significantly increased (e, g), respectively. In addition, the results of Western blot analysis (h) showed that the protein expression of E-cadherin was significantly increased (i) but that of N-cadherin was significantly decreased (j) in the hsa_circ_0000511 silenced group. Mean ± SD; the data were analyzed by an unpaired two-sided *t*-test; vs. the control, ^∗∗^*p* < 0.01 and ^∗∗∗^*p* < 0.001; vs. si-circ-NC, ^##^*p* < 0.01 and ^###^*p* < 0.001.

To study whether hsa_circ_0000511 plays a role in the process of cervical cancer, we silenced hsa_circ_0000511 in vitro through transfecting SiHa and HeLa cells with hsa_circ_0000511 silenced plasmid. Firstly, we analyzed this effect on the proliferation of SiHa and HeLa cells. The results showed that the absorbances of the si-circ group were significantly higher than those of the control group and si-circ-NC group (Figures 2(a) and 2(b)). Meanwhile, the results of qRT-PCR showed that the relative expressions of hsa_circ_0000511 in the si-circ group were obviously lower than those in the control group and si-circ-NC group, respectively (Figure 2(c)). Next, we investigated the effect of hsa_circ_0000511 silencing on cell invasion and migration (Figures 2(d) and 2(f)). The results showed that the numbers of invasive or migratory cells in the si-circ group were significantly higher than that in the control group and si-circ-NC group, respectively (Figures 2(e) and 2(g)). Epithelial mesenchymal transition (EMT) is an important factor leading to the functional changes of cell migration and invasion [26], and the abnormal expression of N-cadherin and E-cadherin is considered a hallmark of EMT [27]. Therefore, we continue to study the effect of hsa_circ_0000511 silencing on the expression of E-cadherin and N-cadherin. As shown in Figures 2(h)–2(j), the protein expressions of E-cadherin in the si-circ group were significantly higher than those in the control group and si-circ-NC group, while those of N-cadherin in the si-circ group were evidently lower than those in the control group and si-circ-NC group.

### 3.3. hsa_circ_0000511 Promoted the Invasion and Migration of SiHa and HeLa Cells by Sponging hsa-miR-296-5p

**Figure 3:**
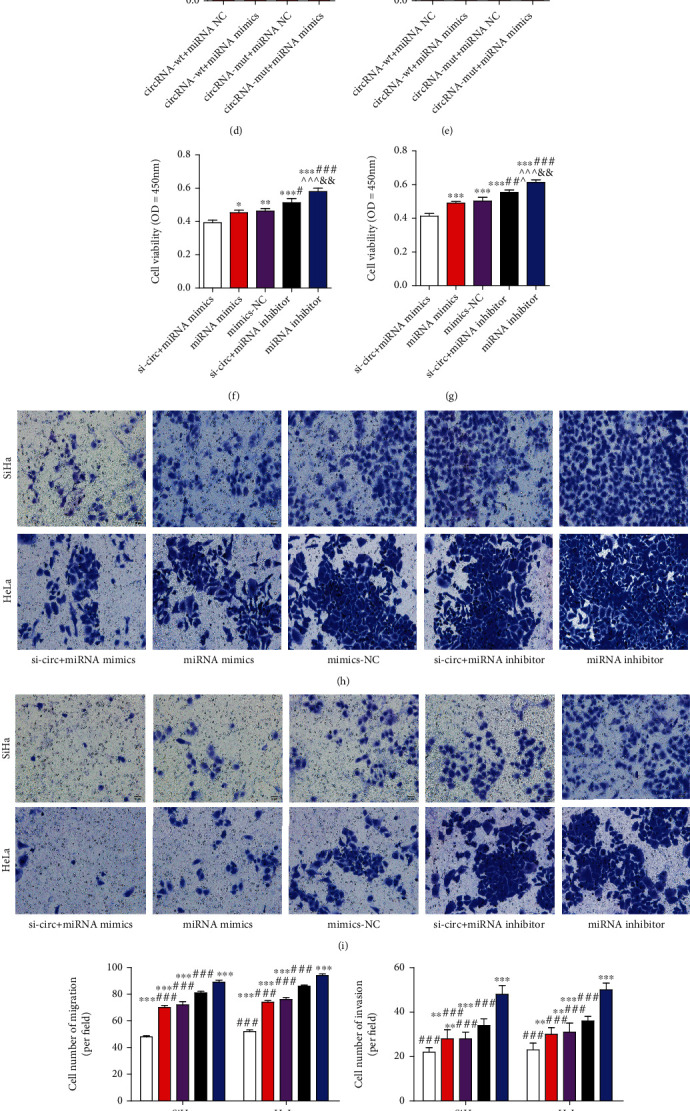
hsa_circ_0000511 promoted the invasion and migration of SiHa and HeLa cells by regulating hsa-miR-296-5p. Contrary to hsa_circ_0000511, the expression of hsa-miR-296-5p in SiHa and HeLa cells was significantly lower than that in H8 and C-33A cells (a) (mean ± SD; the data were analyzed by one-way analysis of variance, vs. H8, ^∗∗^*p* <0.01; vs. C-33A, *^##^p* <0.01), and that was increased when hsa_circ_0000511 was silenced (b) (mean ± SD, the data were analyzed by an unpaired two-sided *t*-test, vs. the control, ^∗∗^*p* < 0.01; vs. si-circ-NC, ^##^*p* < 0.01). The result of dual-luciferase reporter assay (c) shows that hsa-miR-296-5p is a direct target of hsa_circ_0000511 in SiHa (d) and HeLa (e) cells (mean ± SD; the data were analyzed by one-way analysis of variance; vs. circRNA-wt+miRNA NC, ^∗∗^*p* < 0.01). The proliferation (f, g), invasion (h), and migration (i) of SiHa and HeLa cells were increased because hsa-miR-296-5p was regulated by hsa_circ_0000511; that is to say, the number of cells in a lower Transwell chamber was increased significantly (j, k). In addition, the expression of hsa-miR-296-5p (l) changed when whether hsa_circ_0000511 (m) was silenced or not. (f, g, j–m) Mean ± SD; the data were analyzed by one-way analysis of variance; vs. si-circ+miRNA mimics, ^∗^*p* < 0.05, ^∗∗^*p* < 0.01, and ^∗∗∗^*p* < 0.001; vs. miRNA mimics, ^#^*p* < 0.05, ^##^*p* < 0.01, and ^###^*p* < 0.001; vs. mimics NC, ^^^*p* < 0.05, ^^^^*p* < 0.01, and ^^^^^*p* < 0.001; vs. si-circ+miRNA inhibitor, ^&&^*p* < 0.01 and ^&&&^*p* < 0.001.

To explore the potential molecular mechanism of hsa_circ_0000511 promoting EMT, we used online databases of circBank, Circinteractome, and starBase to predict the potential miRNA target of hsa_circ_0000511 and found that hsa-miR-296-5p was a target of hsa_circ_0000511. To verify it, we first found that the expressions of hsa-miR-296-5p in SiHa and HeLa cells were significantly lower than those in H8 and C-33A cells (Figure 3(a)), and its expression in the si-circ group was significantly higher than that in the control group and si-circ-NC group (Figure 3(b)). Then, we performed a dual-luciferase reporter assay to confirm whether hsa_circ_0000511 directly interacts with hsa-miR-296-5p (Figure 3(c)). The results show that the luciferase activity of the circRNA (wt)+miRNA mimics group was obviously lower than that of the circRNA (wt)+miRNA NC group, while that of the circRNA (mut)+miRNA mimics group did not obviously change compared to that of the circRNA (mut)+miRNA NC group (Figures 3(d) and 3(e)).

Then, we continued to study the effect of hsa_circ_0000511 and hsa-miR-296-5p interaction on cells and found that the cell viability of the miRNA inhibitor group was significantly lower than that of other groups, and the effect of hsa-miR-296-5p on cell viability was dependent on the effect of hsa_circ_0000511 (Figures 3(f) and 3(g)). Subsequently, we used Transwell assay to detect the effect of their interaction on the invasion and migration of SiHa and HeLa cells. The results showed that the number of invasive and migratory cells decreased significantly in the si-circ+miRNA mimics group. Although the number of invasive or migratory cells in the miRNA mimics group, miRNA NC group, si-circ+miRNA inhibitor group, and miRNA inhibitor group was significantly higher than that in the si-circ+miRNA mimics group, the number of invasive and migratory cells in the miRNA inhibitor group was significantly higher than that in the other three groups (Figures 3(h)–3(k)). Meanwhile, the results of qRT-PCR showed that the expression of hsa_circ_0000511 in the miRNA mimics group, miRNA NC group, and miRNA inhibitor group was obviously higher than that in the si-circ+miRNA mimics group and si-circ+miRNA inhibitor group, while the expression of hsa-miR-296-5p in the si-circ+miRNA mimics group and miRNA mimics group was markedly higher than that in the miRNA NC group, si-circ+miRNA inhibitor group, and miRNA inhibitor group (Figures 3(l) and 3(m)).

### 3.4. hsa-miR-296-5p Inhibited the Invasion and Migration of SiHa and HeLa Cells by Downregulating HMGA1

**Figure 4:**
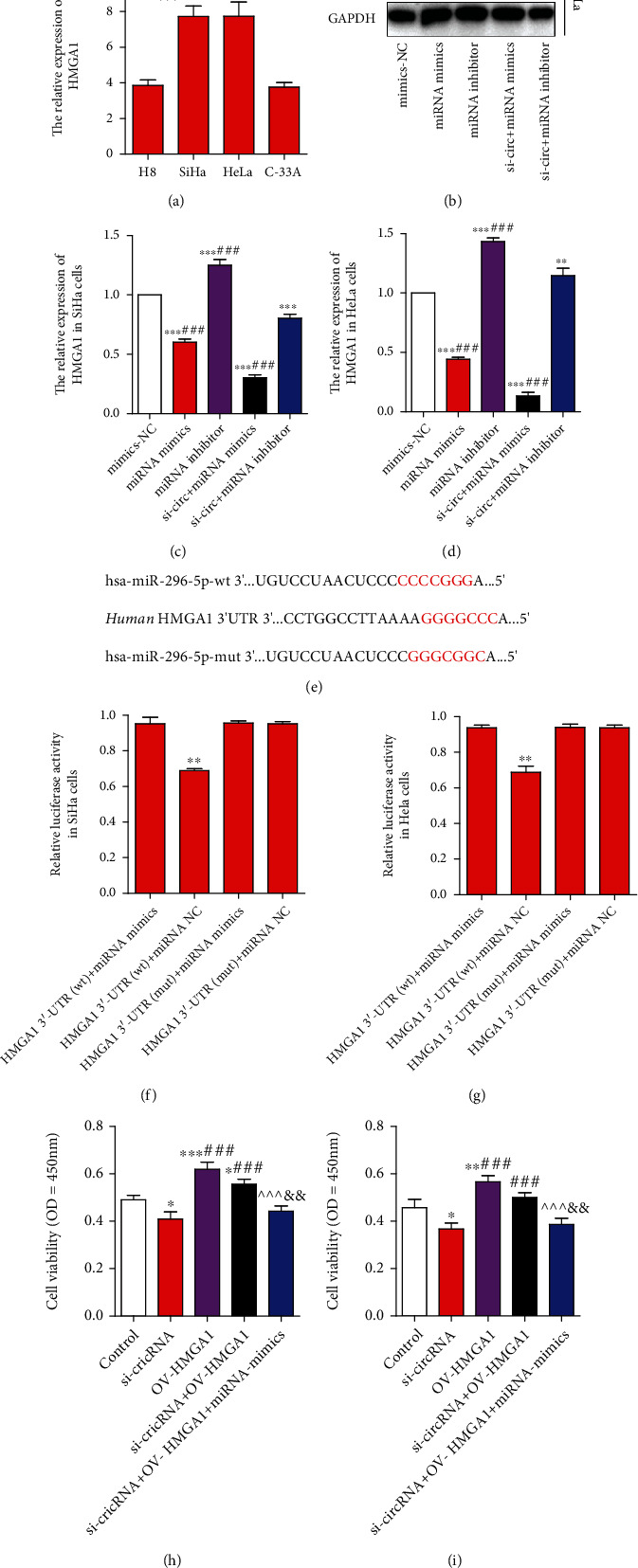
HMGA1 is a direct target of hsa-miR-296-5p, and overexpression of HMGA1 can promote proliferation of SiHa and HeLa cells. Like hsa_circ_0000511, HMGA1 was highly expressed in SiHa and HeLa cells than in H8 and C-33A cells (a) (mean ± SD; the data were analyzed by one-way analysis of variance; vs. H8, ^∗∗^*p* < 0.01; vs. C-33A, *^##^p* < 0.01). The results of Western blot (b) showed that the protein expressions of HMGA1 were changed when hsa-miR-296-5p was inhibited or not in SiHa (c) and HeLa (d) cells, and a dual-luciferase reporter assay (e) indicated that HMGA1 is a direct target of hsa-miR-296-5p ((c, d) mean ± SD, the data were analyzed by one-way analysis of variance; vs. mimics-NC, ^∗∗^*p* < 0.01 and ^∗∗∗^*p* < 0.001; vs. si-circ+miRNA inhibitor, ^###^*p* < 0.001; (f, g), vs. HMGA1 3′-UTR (wt)+miRNA NC, ^∗∗^*p* < 0.01). Moreover, CCK8 detection shown that overexpression of HMGA1 could promote proliferation of SiHa (h) and HeLa (i) cells. Mean ± SD; the data were analyzed by one-way analysis of variance; vs. the control, ^∗^*p* < 0.05, ^∗∗^*p* < 0.01, and ^∗∗∗^*p* < 0.001; vs. si-circRNA, ^###^*p* < 0.001; vs. OV-HMGA1, ^^^^^*p* < 0.001; vs. si-circRNA+OV-HMGA1+miRNA mimics, ^&&^*p* < 0.01.

To further study the target genes of hsa-mir-296-5p, we used miRTarBase, miRDB, starBase, and TargetScan7.2 to predict, in which miRDB was selected according to the score greater than 80. The results showed that there were two common target genes (NFIC and HMGA1), and HMGA1 was selected as a candidate gene because it was widely studied. To verify it, we also investigated the expression of HMGA1 in different cervical cancer cells and found that the expression level of HMGA1 in SiHa and HeLa cells was higher than that in H8 and C-33A cells (Figure 4(a)). Then, we detected the protein expression of HMGA1 under different conditions of miRNA mimics and miRNA inhibitor. The results showed that the expression of HMGA1 was downregulated under the action of miRNA mimics but upregulated when miRNA was inhibited (Figures 4(b)–4(d)), which suggested that hsa-mir-296-5p may have a direct interaction with HMGA1. To further confirm it, a dual-luciferase reporter assay was performed (Figure 4(e)) and the results showed that the luciferase activity of the HMGA1 3′-UTR (wt)+miRNA mimics group was lower than that of the HMGA1 3′-UTR+miRNA NC, and there was no significant difference in the luciferase activity between the HMGA1 3′-UTR (mut)+miRNA mimics and HMGA1 3′-UTR (mut)+miRNA NC groups (Figures 4(f) and 4(g)).

**Figure 5:**
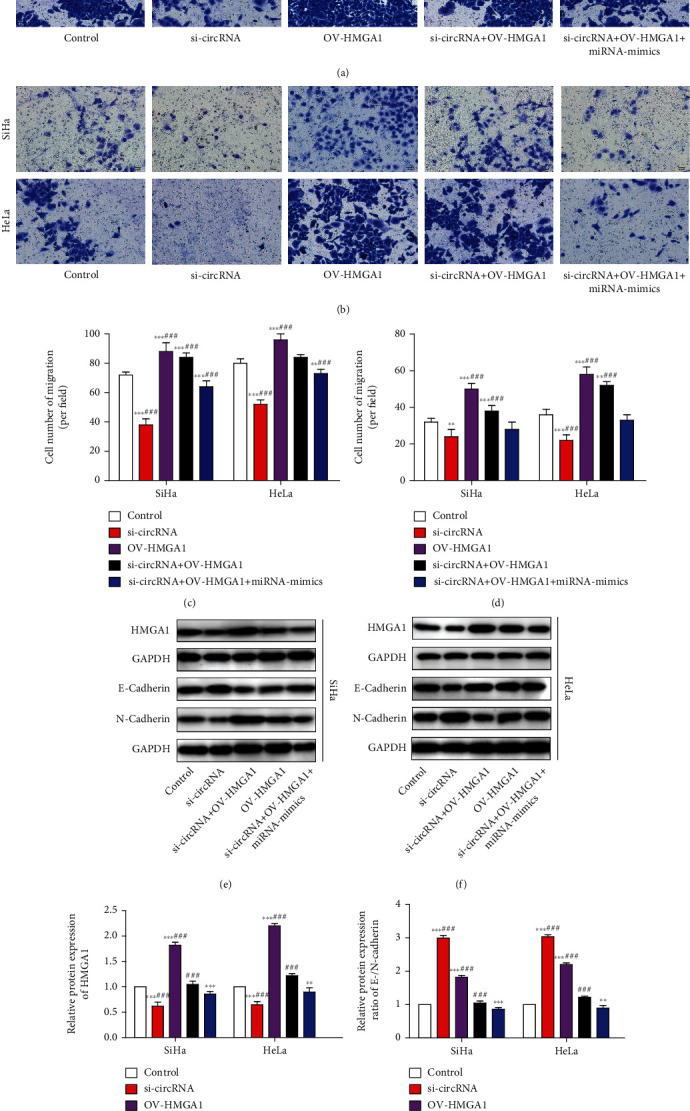
hsa_circ_0000511 promoted EMT by regulating hsa-miR-296-5p/HMGA1 signal. Overexpression of HMGA1 increased the ability of invasion (a) and migration (b) in SiHa and HeLa cells, and the number of cells in the lower Transwell chamber was increased significantly (c, d). Further, the results of Western blotting (e, f) indicated that the expression of E-cadherin and N-cadherin (h) was consistent with that of HMGA1 (g). Overexpressed HMGA1 inhibited the protein expression of E-cadherin and promoted that of N-cadherin, and vice versa. Mean ± SD; the data were analyzed by one-way analysis of variance; vs. control, ^∗∗^*p* < 0.01 and ^∗∗∗^*p* < 0.001; vs. si-circRNA+OV-HMGA1+miRNA mimics, ^###^*p* < 0.001.

Next, we continue to analyze the effects of this regulatory mechanism on cell proliferation, invasion, migration, and EMT of SiHa and HeLa cells. The results of CCK-8 assay showed that overexpression of HMGA1 could significantly promote the proliferation of SiHa and HeLa cells, but this effect could be inhibited by miRNA mimics (Figures 4(h) and 4(i)). Similar results were obtained in Transwell assay, the number of invasive or migratory cells in the si-circRNA+OV-HMGA1 and OV-HMGA1 groups was obviously higher than that in the control or si-circRNA group, but that in the si-circRNA+OV-HMGA1+miRNA-mimics group was lower than that in the si-circRNA+OV-HMGA1 or OV-HMGA1 group (Figure 5(a)–4(d)). Meanwhile, we analyzed the expression of HMGA1 and EMT-related proteins by Western blot. The results showed that the protein expression ratio between E-cadherin and N-cadherin was significantly decreased when HMGA1 was highly expressed, while the protein expression ratio of E/N-cadherin was significantly increased when HMGA1 was expressed low (Figures 5(e)–5(h)).

### 3.5. hsa_circ_0000511 Promotes EMT of Cervical Cancer Cells In Vivo by Downregulating hsa-miR-296-5p/HMGA1

**Figure 6:**
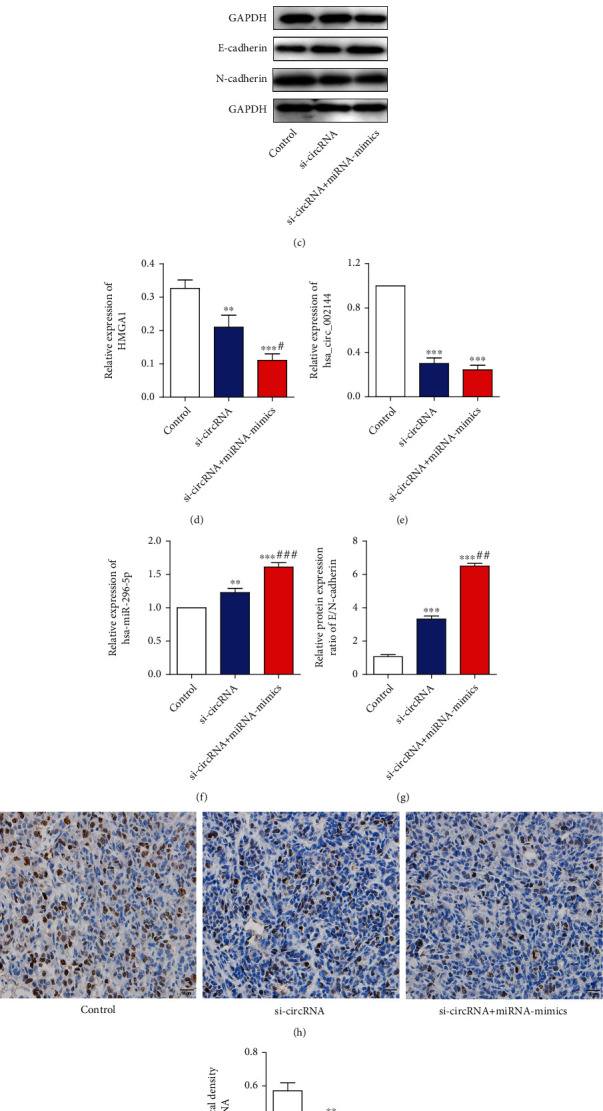
hsa_circ_0000511 promotes EMT of cervical cancer cells in vivo by downregulating hsa-miR-296-5p/HMGA1. Inhibition of hsa_circ_0000511 significantly reduced the tumor volume in vivo (a, b), and Western blot analysis (c) showed that the protein expression of HGMA1 was decreased in turn when hsa_circ_0000511 was silenced or hsa-miR-296-5p was activated (d), while the protein expression ration between E-cadherin and N-cadherin was opposite to that of HGMA1 (e). Meanwhile, the results of qRT-PCR showed that the expression of hsa_circ_0000511 was consistent with the protein expression of HGMA1 (f) but the expression of hsa-miR-296-5p (g) was opposite to that of hsa_circ_0000511. Besides, changes of average optical density of PCNA (h, i) were consistent with the expression of hsa_circ_0000511. Mean ± SD; the data were analyzed by one-way analysis of variance; vs. the control, ^∗∗^*p* < 0.01 and ^∗∗∗^*p* < 0.001; vs. si-circRNA, ^#^*p* < 0.05, ^##^*p* < 0.01, and ^###^*p* < 0.001.

To further confirm the results of our in vitro experiments, we injected the transfected SiHa and HeLa cells subcutaneously into nude mice to observe the tumor growth. The results showed that the tumor weight of the si-circRNA+miRNA mimics group was significantly lower than that of the control group and si-circRN group (Figures 6(a) and 6(b)). The results of Western blot analysis were consistent with the results in vitro, that is, the expression ratio of E/N-cadherin protein was significantly increased when HMGA1 expression was low (Figures 6(c)–6(e)). Meanwhile, the results of qRT-PCR showed the expressions of hsa_circ_0000511 in the si-circRNA group and si-circRNA+miRNA mimics group were significantly higher than that in the control group, while the expression of hsa-mir-296-5p was opposite to that of hsa_circ_0000511 and that in si-circRNA+miRNA mimics group was higher than that in other two groups (Figures 6(f) and 6(g)). In addition, the protein expression of PCNA in tumor was detected by immunohistochemistry (Figure 6(h)) and the results showed that the average optical density of PCNA in the si-circRNA group and si-circRNA+miRNA mimics group was significantly lower than that in control group, while that in the si-circRNA+miRNA mimics group was lower than that in the si-circRNA group (Figure 6(i)).

## 4. Discussion

With the wide application of high-throughput sequencing and microarray analysis, more and more attention has been paid to the important role of circRNAs in the progression of cancer. Only in cervical cancer, dozens of circRNAs have been reported, such as circPCNX [28], circAMOTL1 [12], circCDKN2B-AS1 [29], and circNFATC3 [13]. In a recent study, 353 of 9359 circRNAs were found to be differentially expressed between the cervical cancer group and normal group, and 881 mRNA transcripts were differentially expressed [30]. In the study, we found a highly upregulated circRNA (circ_0000511) in a variety of cervical cancer cells by GEO analysis (GSE113696) and it promoted the proliferation of SiHa and HeLa cells when it was silenced. Further, we found it can promote cell proliferation, invasion, and metastasis of SiHa and HeLa cells through regulating hsa-miR-296-5p/HMGA1, which is related to the regulation of EMT in vitro and in vivo experiments. The study indicated that the hsa_circ_0000511/hsa-miR-296-5p/HMGA1 axis may play an important role in the development of cervical cancer.

EMT, a cellular process in which epithelial cells acquire mesenchymal phenotype and behavior after epithelial characteristics are down regulated, is triggered by signals received by cells from a microenvironment [26]. EMT cells not only show fibroblast like morphology and cytoarchitecture but also increase the capacity of migration and invasiveness [26]. It is noteworthy that the progression of EMT is characterized by the changes in gene expression and the posttranslational regulatory mechanisms of multiple connections to epithelial cells, including the adherens junctions, desmosomes, gap junctions, and tight junctions [26]. E-cadherin belongs to type I classic cadherin, which plays an important role in maintaining epithelial phenotype and regulating tissue homeostasis [31]. Studies have found that deficiency of E-cadherin in cancer cells causes the metastasis and activation of a variety of EMT transcription factors [32]. Since the recovery of E-cadherin expression in E-cadherin-negative malignant cells can not reverse EMT, some researchers do not agree that the decrease of E-cadherin expression is a marker of EMT [33]. At the same time, various invasive and metastatic cancers such as prostate cancer, ovarian cancer, and glioblastoma are associated with high levels of E-cadherin expression [27]. However, the dual effects of E-cadherin may be due to the existence of membrane tethered E-cadherin and soluble E-cadherin [34]. Although both E-cadherin and N-cadherin belong to type I classic cadherin, N-cadherin can embed endothelial cells and parietal cells to stabilize microvessels and then promote angiogenesis and maintain vascular integrity [27]. Thus, it is considered an indicator of ongoing EMT, and a large number of studies have confirmed that its expression is related to the development of various types of cancer [27]. In addition, studies have shown that E-cadherin mainly inhibits the activation of the Wnt/*β*-catenin and RTK/P13K pathway in epithelial cells, while N-cadherin-mediated adhesion promotes the activation of the MAPK/ERK signal and PI3K/PDGFR pathway to enhance cell survival and migration in nonepithelial cells [27]. So the abnormal expression of N-cadherin and E-cadherin is considered a hallmark of EMT.

HMGA1, belonging to the superfamily of nonhistone chromatin binding proteins, is a new transcription regulator and involved in a variety of cellular biological processes, including transcriptional regulation, DNA repair, cell cycle regulation, differentiation, embryogenesis, transformation, and viral integration [35]. Numerous studies have found that it was highly expressed in different cancers, including cervical cancer [36, 37], gastric cancer [38, 39], liver cancer [40], renal cancer [41], and other cancers [35]. Moreover, HMGA1 has been found to amplify Wnt signal and maintain Wnt and other transcriptional networks, suggesting that HMGA1 overexpression is involved in tumorigenesis and progression through dysregulation [25]. Significantly, HMGA1 has been confirmed to be regulated by miRNA such as miR-125b [42], miR-221/222 [37], miR-296-5p [36], and miR-214 [43] and was involved in EMT. In the study, we found that miR-296-5p can mediate HMGA1 to regulate EMT in SiHa and HeLa cells; it also confirmed that HMGA1 is a direct target of miR-296-5p [36]. In addition, we also proved that miR-296-5p is regulated by hsa_circ_0000511 and may also participate in regulating PCNA, but its molecular mechanism needs further study.

Summary, we proved that silenced hsa_circ_0000511 reduces the proliferation and EMT of SiHa and HeLa cells through inhibiting the expression of HMGA1 by upregulating miR-296-5p. It is worth noting that we observed the invasion and migration of cells in this study, but only investigated the proteins related to adhesion, not degradation and migration-related proteins. At the same time, we predicted that hsa-mir-296-5p had two common target genes (NFIC and HMGA1), but we only verified HMGA1. We will carry on the corresponding research to these contents in the future. The study may contribute to understand the molecular mechanism of cell invasion and migration in the process of cervical cancer and provide a potential therapeutic target.

## Figures and Tables

**Table 1:** Primer sequence.

Primer name	Primer sequence
hsa_circ_0000511 F	5′-GAACAGACTCACGGCCAGCGAAGTGAGTTC-3′
hsa_circ_0000511 R	5′-GAACTCACTTCGCTGGCCGTGAGTCTGTTC-3′
*RPPH1* human F [23]	5′-GAGCTGAGTGCGTCCTGTC-3′
*RPPH1* human R	5′-TCAGGGAGAGCCCTGTTAGG-3′
miR-296-5p F [24]	5′-CCCCCCCTCAATCCTGT-3′
miR-296-5p R	5′-CAACTGGTGTCGTGGAG-3′
HMGA1 F [25]	5′-GGCCCAAATCGACCATAAAGG-3′
HMGA1 R	5′-GGACAAATCATGGCTACCCCT-3′
GAPDH F [23]	5′-CAGCCTCAAGATCATCAGCA-3′
GAPDH R	5′-TGTGGTCATGAGTCCTTCCA-3′

**Table 2:** The sequence of primer for siRNA.

Primer name	Primer sequence
siRNA	5′-CUGGCCAAGGGGCCUUUACATT-3′
si-negative control	5′-UUCUUCGAACGUGUCACGUTT-3′
Mimics-NC	5′-UUCUCCGAACGUGUCACGUTT-3′
miR-296-5p mimics	5′-UGUCCUAACUCCCCCCCGGGA-3′
Inhibitor-NC	5′-CAGUACUUUUGUGUAGUACAA-3′
miR-296-5p inhibitor	5′-UCCCGGGGGGGAGUUAGGACA-3′

## Data Availability

The datasets and supporting data are available from the corresponding author upon reasonable request.

## References

[1] Arbyn M., Weiderpass E., Bruni L. (2020). Estimates of incidence and mortality of cervical cancer in 2018: a worldwide analysis.. *The Lancet Globalization and Health*.

[2] Stelzle D., Tanaka L. F., Lee K. K. (2020). Estimates of the global burden of cervical cancer associated with HIV. *The Lancet Global health*.

[3] Lei J., Ploner A., Elfström K. (2020). HPV vaccination and the risk of invasive cervical cancer. *The New England Journal of Medicine*.

[4] Canfell K., Kim J., Brisson M. (2020). Mortality impact of achieving WHO cervical cancer elimination targets: a comparative modelling analysis in 78 low-income and lower-middle-income countries. *Lancet*.

[5] de Sanjose S., Delany-Moretlwe S. (2019). HPV vaccines can be the hallmark of cancer prevention. *Lancet*.

[6] Chen L. L. (2016). The biogenesis and emerging roles of circular RNAs. *Nature Reviews. Molecular Cell Biology*.

[7] Han B., Chao J., Yao H. (2018). Circular RNA and its mechanisms in disease: from the bench to the clinic. *Pharmacology & Therapeutics*.

[8] Panda A. C., Grammatikakis I., Kim K. M. (2017). Identification of senescence-associated circular RNAs (SAC-RNAs) reveals senescence suppressor CircPVT1. *Nucleic acids research*.

[9] Chuang T. J., Wu C. S., Chen C. Y., Hung L. Y., Chiang T. W., Yang M. Y. (2016). NCLscan: accurate identification of non-co-linear transcripts (fusion, trans-splicing and circular RNA) with a good balance between sensitivity and precision. *Nucleic Acids Research*.

[10] Du W. W., Fang L., Yang W. (2017). Induction of tumor apoptosis through a circular RNA enhancing Foxo3 activity. *Cell Death and Differentiation*.

[11] Jeck W. R., Sorrentino J. A., Wang K. (2013). Circular RNAs are abundant, conserved, and associated with ALU repeats. *Rna*.

[12] Sun Z., Niu S., Xu F., Zhao W., Ma R., Chen M. (2020). CircAMOTL1 promotes tumorigenesis through miR-526b/SIK2 axis in cervical cancer. *Frontiers in cell and developmental biology*.

[13] Ma N., Li X., Wei H., Zhang H., Zhang S. (2020). Circular RNA circNFATC3 acts as a miR-9-5p sponge to promote cervical cancer development by upregulating SDC2. *Cellular Oncology (Dordr)*.

[14] Li X., Ma N., Zhang Y. (2020). Circular RNA circNRIP1 promotes migration and invasion in cervical cancer by sponging miR-629-3p and regulating the PTP4A1/ERK1/2 pathway. *Cell Death & Disease*.

[15] Ou R., Mo L., Tang H. (2020). circRNA-AKT1 sequesters miR-942-5p to upregulate AKT1 and promote cervical cancer progression. *Molecular therapy Nucleic acids*.

[16] Ou R., Lv J., Zhang Q. (2020). circAMOTL1 motivates AMOTL1 expression to facilitate cervical cancer growth. *Molecular therapy Nucleic acids*.

[17] Jiao J., Zhang T., Jiao X. (2020). hsa_circ_0000745 promotes cervical cancer by increasing cell proliferation, migration, and invasion. *Journal of Cellular Physiology*.

[18] Cai H., Zhang P., Xu M., Yan L., Liu N., Wu X. (2019). Circular RNA hsa_circ_0000263 participates in cervical cancer development by regulating target gene of miR-150-5p. *Journal of Cellular Physiology*.

[19] Hu C., Wang Y., Li A., Zhang J., Xue F., Zhu L. (2019). Overexpressed circ_0067934 acts as an oncogene to facilitate cervical cancer progression via the miR-545/EIF3C axis. *Journal of cellular physiology*.

[20] Lee Y. S., Dutta A. (2009). MicroRNAs in cancer. *Annual Review of Pathology*.

[21] Nahand J., Taghizadeh-boroujeni S., Karimzadeh M. (2019). microRNAs: new prognostic, diagnostic, and therapeutic biomarkers in cervical cancer. *Journal of Cellular Physiology*.

[22] Zhang J., Cai R., Zhang Y., Wang X. (2020). Involvement of a novel circularRNA, hsa_circ_0000520, attenuates tumorigenesis of cervical cancer cell through competitively binding with miR-146b-3p. *Journal of Cellular and Molecular Medicine*.

[23] Cai Y., Sun Z., Jia H. (2017). Rpph1 upregulates CDC42 expression and promotes hippocampal neuron dendritic spine formation by competing with miR-330-5p. *Frontiers in molecular neuroscience*.

[24] Li M. M., Liu X. H., Zhao Y. C. (2020). Long noncoding RNA KCNQ1OT1 promotes apoptosis in neuroblastoma cells by regulating miR-296-5p/Bax axis. *The FEBS Journal*.

[25] Resar L., Chia L., Xian L. (2018). Lessons from the crypt: HMGA1-amping up Wnt for stem cells and tumor progression. *Cancer Research*.

[26] On behalf of the EMT International Association (TEMTIA), Yang J., Antin P. (2020). Guidelines and definitions for research on epithelial-mesenchymal transition. *Nature Reviews Molecular Cell Biology*.

[27] Loh C., Chai J., Tang T. (2019). The E-cadherin and N-cadherin switch in epithelial-to-mesenchymal transition: signaling, therapeutic implications, and challenges. *Cell*.

[28] Tsitsipatis D., Grammatikakis I., Driscoll R. (2021). AUF1 ligand circPCNX reduces cell proliferation by competing with p21 mRNA to increase p21 production. *Nucleic Acids Research*.

[29] Zhang Y., Zhao L., Yang S. (2020). CircCDKN2B-AS1 interacts with IMP3 to stabilize hexokinase 2 mRNA and facilitate cervical squamous cell carcinoma aerobic glycolysis progression. *Journal of experimental & clinical cancer research*.

[30] Xu T., Song X., Wang Y., Fu S., Han P. (2020). Genome-wide analysis of the expression of circular RNA full-length transcripts and construction of the circRNA-miRNA-mRNA network in cervical cancer. *Frontiers in cell and developmental biology*.

[31] van Roy F., Berx G. (2008). The cell-cell adhesion molecule E-cadherin. *Cellular and Molecular Life Sciences*.

[32] onder T. T., Gupta P. B., Mani S. A., Yang J., Lander E. S., Weinberg R. A. (2008). Loss of E-cadherin promotes metastasis via multiple downstream transcriptional pathways. *Cancer Research*.

[33] Hollestelle A., Peeters J. K., Smid M. (2013). Loss of E-cadherin is not a necessity for epithelial to mesenchymal transition in human breast cancer. *Breast Cancer Research and Treatment*.

[34] Hu Q. P., Kuang J. Y., Yang Q. K., Bian X. W., Yu S. C. (2016). Beyond a tumor suppressor: soluble E-cadherin promotes the progression of cancer. *International Journal of Cancer*.

[35] Zhong J., Liu C., Zhang Q. H. (2017). TGF-*β*1 induces HMGA1 expression: the role of HMGA1 in thyroid cancer proliferation and invasion. *International Journal of Oncology*.

[36] Ma W. G., Shi S. M., Chen L., Lou G., Feng X. L. (2021). SP1-induced lncRNA FOXD3-AS1 contributes to tumorigenesis of cervical cancer by modulating the miR-296-5p/HMGA1 pathway. *Journal of Cellular Biochemistry*.

[37] Fu F., Wang T., Wu Z. (2018). HMGA1 exacerbates tumor growth through regulating the cell cycle and accelerates migration/invasion via targeting miR-221/222 in cervical cancer. *Cell Death & Disease*.

[38] Jin G. H., Shi Y., Tian Y., Cao T. T., Mao Y., Tang T. Y. (2020). HMGA1 accelerates the malignant progression of gastric cancer through stimulating EMT. *European Review for Medical & Pharmacological Sciences*.

[39] Wang J., Lv B., Su Y., Wang X., Bu J., Yao L. (2019). Exosome-mediated transfer of lncRNA HOTTIP promotes cisplatin resistance in gastric cancer cells by regulating HMGA1/miR-218 axis. *Oncotargets and Therapy*.

[40] Teng K., Wei S., Zhang C. (2019). KIFC1 is activated by TCF-4 and promotes hepatocellular carcinoma pathogenesis by regulating HMGA1 transcriptional activity. *Journal of Experimental & Clinical Cancer Research*.

[41] Dong H., Sun S., Yan T. (2020). MicroRNA-195 inhibits proliferation and metastasis in renal cell carcinoma via regulating HMGA1. *American Journal of Translational Research*.

[42] Sun B., Zhang Y., Zhou L. (2019). The proliferation of cervical cancer is promoted by miRNA-125b through the regulation of the HMGA1. *OncoTargets and therapy*.

[43] Chandrasekaran K. S., Sathyanarayanan A., Karunagaran D. (2017). miR-214activates TP53 but suppresses the expression of RELA, CTNNB1, and STAT3 in human cervical and colorectal cancer cells. *Cell Biochemistry and Function*.

